# Supplementary material for: Health care providers’ knowledge and associated factors towards the components of post abortion care service in hospitals of Tigray, Northern Ethiopia
Source: PLOS Glob Public Health. 2025 Apr 24;5(4):e0004412. doi: 10.1371/journal.pgph.0004412 (PMC12021144; doi:10.1371/journal.pgph.0004412)
Supplement: S1 File — (PDF) [file pgph.0004412.s002.pdf]

Health care providers' knowledge and associated factors towards the components of post abortion care service in hospitals of Tigray, Northern Ethiopia.

Questionnaires

Annex II

Part I. Socio demographic characteristics

Direction: choose one possible answers and circle it.

Number	Questions	Responses options	Skip
Socio-demographic variables			
1.	Age	________Year	
2.	Gender	1. Male 2. Female	
3.	ethnicity	1.Tigray 3.Oromo<br>2.Amahara 4.Others [Specify)	
4.	What is your Religion?	1. Orthodox 3. Muslim<br>2. Catholic 4.Protestant	
5.	What is your current Marital status?	1. Single 4. Cohabit<br>2. Married 5. Divorced<br>3. Widow	
6.	What is your educational level	1. Diploma 3.Master and above<br>2.Degree	

Part II: job and setup related variables

	Questions	Response option	Skip
7	What is your profession?	1. Midwifery 4. Nurse<br>2. Medical doctor 5. HO<br>3. IESO	
8	Years of experience	…………………..	

9	Where do you work?	1. General Hospital 2. Referral hospital	
10	Have you ever attended abortion care?	1. Yes, 2. No	
11	Have you ever trained for post-abortion care?	1. Yes, 2. No	If no 14
12	If yes When you trained?	………………….	
13	If yes for Q 11, are you practicing it now?	1.Yes 2. No	

14	Is there any responsible body who regularly Supervised your work?	1. Yes 2. No	
15	If yes how often do they supervise the clinics?		
16	Do you have a written guideline for post-abortion care?	1.Yes 2. No	
17	If yes, are you using it now?	1.Yes 2. No	
18	If No for Q 16, why?		
19	Do you have a reference site (reading room) for abortion management in your hospital?	1. Yes, 2. No	
20	Do you gain on job training for PAC?	1. Yes, 2. No	
21	Which drug/drugs is/are available for abort pregnancy?		
22	Is there available of contraceptives at the site was the PAC procedure is offered?	1.Yes 2.No	
23	How many clients on average attend the clinic per day?	-----------	
24	How many professions do you work in one shift?	-------------	
25	Are you motivated to read about abortion care?		

Part III. Knowledge related questions on components of post-abortion care.

26	Could you mention the components of post-abortion care?	Listed	not listed	
28	Emergency treatment			
29	Counseling			
30	Family planning			
31	Linkage to RH and other health services			

32	Community and service partnership				
