# Supplementary material for: Health care providers’ knowledge and associated factors towards the components of post abortion care service in hospitals of Tigray, Northern Ethiopia
Source: PLOS Glob Public Health. 2025 Apr 24;5(4):e0004412. doi: 10.1371/journal.pgph.0004412 (PMC12021144; doi:10.1371/journal.pgph.0004412)
Supplement: Attachment — Submitted filename: response to reviewers.pdf [file pgph.0004412.s004.pdf]

Subject: Revised Manuscript Submission – Response to Reviewer Comments

Dear Editors of PLOS Global Public Health,

I hope this message finds you well.

We, the authors of the manuscript entitled "Healthcare Providers' Knowledge and Associated Factors Towards the Components of Post-Abortion Care Services in Hospitals of Tigray, Northern Ethiopia" (PGPH-D-24-02390), would like to express our sincere gratitude for the constructive and invaluable feedback provided by the reviewers. We greatly appreciate the time and effort they have dedicated to reviewing our manuscript, and we believe their comments have significantly contributed to the improvement and strengthening of our work.

In response to the reviewer comments, we have thoroughly revised the manuscript, addressing each point raised. A detailed point-by-point response has been provided to ensure clarity and to demonstrate how each comment has been incorporated. We believe that these revisions have enhanced the quality and rigor of the manuscript.

We have now submitted the revised version of the manuscript along with all the required documents. We trust that we have adequately addressed all concerns, but should you or the reviewers require further clarification or additional information, please do not hesitate to reach out.

Once again, we would like to extend our sincere thanks to the editorial team and the reviewers for their invaluable feedback and support.

Regards,

Gidey Goitom

On behalf of all authors

gideygoitom34@gmail.com

Reviewer comments:

Reviewer 1.

Abstract

Make the abstract more specific and concise. Include only important information in the abstract and avoid jargon. For example, in the background you described about the burden associated with complications of pregnancy and child birth and included information about the magnitude of unsafe abortion which are not specific for the topic. Please try to focus on the magnitude of the problem (knowledge of HP's on the CPAC), what is unknown about the problem (knowledge gap), and what you are going to do about it (objective).

Response: Thank you for your valuable feedback. We have revised the abstract to address your concerns by making it more concise and focused. Specifically, we have removed the broader background information about pregnancy complications and unsafe abortion, and instead emphasized the magnitude of the knowledge gap regarding healthcare professionals' understanding of the CPAC. (please see line 22-29)

Background

Please revise your background based on the above comment. It lacks specificity and coherence.

- Try to discuss about the components of post abortion care in more detail. Define each component and reason out how the knowledge gap specific to each component affect the outcome of abortion care.

Response: Thank you for your constructive feedback and suggestions. We have revised the background section to address your concerns. In the updated version, we provide a more detailed discussion of the components of post-abortion care, defining each element clearly. Additionally, we have elaborated on how the knowledge gap in each component may affect the outcomes of abortion care, as per your recommendation. (please go to line 55-57, line 61-64 and line 66-84)

- Line 54… 'This contributes to 520/100,000 cases of maternal deaths…' is that per women of child bearing age or cases of maternal death? Please check.

Response: Thank you for pointing this out. We have reviewed the statement and clarified the reference to maternal deaths. The figure "520/100,000" refers to the maternal mortality ratio per 100,000 women of reproductive age, not the total number of cases of maternal death. (see line 57-58)

Line 77 '… (CPAC) practices have become common among healthcare providers…' how can you justify this statement? common CPAC practices but low knowledge

Response: Thank you for your insightful comment. While CPAC practices are increasingly implemented among healthcare providers, it is important to note that they are not always

practiced comprehensively. Often, healthcare providers may neglect one or more of the five essential CPAC. This lack of full implementation can significantly impact patient outcomes. For example, performing MVA is a common practice among health care providers however, the complete implementation of PAC components is unlikely. This could be due to knowledge gap or other lack of facility. We have updated the manuscript to clarify this distinction. (Please see line 95-98)

Methodology

Please revise your sample size calculation. The proportion of knowledge about MVA is only one CPAC but you are trying to study the overall knowledge about the 5 components. In addition, how is the facilities and population comparable with that of the Nigerian study?

Response: Thank you for your questions. We acknowledge your concern, but in the absence of a similar study that assesses overall knowledge, we believe focusing on a single component provides a starting point. This approach allows us to gather preliminary data, which can inform future research that includes a broader scope. Regarding the comparison of facilities and populations with the Nigerian study, we understand the importance of contextual relevance. While there are differences between the two countries, we believe that Nigeria's socio-demographic characteristics and healthcare settings, particularly in terms of being a developing country in Africa, share enough similarities with Ethiopia to justify the comparison. Both countries face comparable challenges in terms of healthcare infrastructure, population demographics, and socio-economic conditions, which makes the use of the Nigerian study as a reference reasonable.

Sampling method: out of 2 referral hospitals and 14 general hospitals, you selected 5 general hospital and 2 referral hospitals. You said you used SRS method, but how do you determine the number of hospitals required for study?

Response: Thank you for your valuable feedback. The number of hospitals selected for the study was determined based on a combination of factors, including available resources, the intended scope of the study, and the goal of ensuring a representative sample. For the general hospitals, we employed Simple Random Sampling (SRS) to select 5 out of the 14 available general hospitals but all (2) referral hospital were included purposively to obtain adequate size sample size and diverse professions. (see line 120-122)

Regarding participant selection, since the level of knowledge of the HP's is affected by their level of professionalism (heterogeneous population), it is better to stratify the participants based on their profession (gynecologist, emergency surgery professional, MW, etc.) during sampling.
Response: Thank you for your insightful comments and suggestions. You are right that the level of knowledge among healthcare professionals may vary depending on their specific profession, given the heterogeneous nature of the population. However, in this study, we aimed to focus on all healthcare professionals who provide abortion services, regardless of their specific profession. The rationale behind this approach is that all professionals involved in providing abortion services are expected to deliver the full range of post-abortion care, which includes key components that are relevant across different specialties.

What are your inclusion and exclusion criteria?
Response: Thank you for your comment. The inclusion criteria for our study were all healthcare providers actively involved in providing abortion services at the Obstetrics and Gynecology (Obs/Gyn) ward. exclusion criteria, we did not set any formal exclusion criteria for this study. All

healthcare providers working in the Obs/Gyn ward and participating in abortion services were considered eligible for inclusion, as we aimed to gather a comprehensive perspective from all involved healthcare providers. (line 126-129)

Data collection instrument: did you use a standard questionnaire to ass knowledge?

Response: Thank you for your question. Yes, we used a structured questionnaire designed specifically for this study to assess knowledge. While it was not a standard, widely-used questionnaire, it was carefully developed based on the essential CPAC to ensure its relevance and accuracy in measuring the knowledge of healthcare providers. We also consulted relevant literature and experts to ensure the validity and reliability of the instrument.

Why didn't you use self-administered questionnaires rather than face to face interview? How did you controlled interviewer bias?

Response: Thank you for your question. We opted for face-to-face interviews instead of self-administered questionnaires because the format of the questionnaire was checklist-based. If administered alone, participants might simply tick all available options, which could lead to inaccurate reporting and not reflect their actual knowledge. In contrast, face-to-face interviews allow for a more accurate assessment of the participants' knowledge, as they can verbally express what they know. To minimize interviewer bias, we ensured that interviewers were trained to ask questions neutrally and consistently, without influencing the responses.

To my knowledge, Health professionals in Ethiopia are trained and practice in English and they are supposed to understand English. Why did you need to translate the questionnaire to Tigrigna?

Response: Thank you for raising this important point. While it is true that English is widely used in the training of health professionals in Ethiopia, we recognize that English is the second

language for many individuals. To ensure that participants fully comprehend the questionnaire and provide accurate responses, we deemed it necessary to translate it into Tigrigna, the local language. This translation was essential to minimize any language barriers and ensure clarity, particularly for participants who may have varying levels of proficiency in English. We believe this approach enhanced the validity of the data collected, as it allowed participants to engage more effectively with the content.

Please provide the questionnaire as a supporting material during submission.

Response: Thank you for your suggestion. We will include the questionnaire as a supporting material when submitting the manuscript.

Operational definitions: what is your reference to define good and poor knowledge? Is it a standard method?

Response: Thank you for your question. The definitions of "good" and "poor" knowledge were based on a set criterion we established for the study, specifically the percentage of correct responses. "Good knowledge' was defined as correctly listing all knowledge related questions of the CPAC, while any score missing one or more knowledge-related questions was categorized as 'poor knowledge." This method is not necessarily a standard approach but was chosen to ensure that healthcare providers demonstrated complete understanding of all essential CPAC, as even one missed component can significantly affect patient outcomes. We have explained this rationale clearly in the manuscript. (line 160-164)

Results:

- Please revise your results part. Stick to the journal's statistical reporting guideline.

Response: Thank you, we have tried to stick to the journal's statistical reporting guideline.

- Please avail the data set associated with the reported data in a public repository or provide as a supporting material during submission.

Response: Thank you for your suggestion regarding the data availability. We ensure that the dataset is available and can access with reasonable request.

Discussion:

- The discussion part is very shallow and full of grammar, spelling, and punctuation error. Please try to discuss the main findings adequately based on your objectives.

Response: Thank you for your valuable feedback. We have carefully revised the discussion section to address the comments and make the necessary improvements, ensuring it is more comprehensive and freer from grammatical and punctuation errors. (please go to line 249-1256, 259-263, 282, 269-272, 274-278, and 282-283)

- What is your recommendation based on your study findings?

Response: Thank you again for your comments, we have added the recommendation as below.

Recommendation

To Tigray Regional Health Bureau:

The Tigray Regional Health Bureau should encourage the sharing of experiences and best practices between referral and general hospitals. This can help bridge knowledge gaps and promote a more uniform standard of care across different healthcare settings.

In addition, it is essential to provide updated training to all healthcare providers who offer abortion care, to ensure they remain well-informed about the latest protocols, best practices, and guidelines.

To Health Workers and Healthcare Facilities:

Healthcare providers and facilities should be encouraged to utilize the guidelines available in their clinics, ensuring that they are consistently followed in the provision of post-abortion care. (see line 305-3015)

- Do you think exclusion of health centers and primary hospitals as the limitation of the study in addition to private clinics?

Response: thank you. Yes, one limitation of this study is the exclusion of health centers, primary hospitals, and private clinics, which are also key providers of post-abortion care services. The study focused on referral and general hospitals, which may not fully represent the range of healthcare facilities involved in providing abortion care across the region.

References:

- Please go through your reference and edit.

Response: Thank you for your feedback, we have reviewed the references.

Reviewer 2 -

Background:

- The background did not mention the comprehensive abortion care concept which is another critical milestone on abortion services.

Response: Thank you for your valuable comment. We have revised the background section to include a discussion on the concept of comprehensive abortion care, as it is indeed a critical milestone in abortion services. (see line 61-64)

Methods:

- The data as well looks a bit older (about 5 years old).

Response: Thank you for your suggestion. While the data may be around five years old, this study focuses on the essential components of post-abortion care, which have not been extensively studied in the Tigray region of Ethiopia. The lack of research in this specific area justifies the continued relevance and importance of this study, regardless of the data's age. We have emphasized this in the manuscript to clarify the rationale behind using this data.

Discussion:

- The discussion section is also abbreviated.

Response: Thank you for your comment. We have carefully revised the discussion section to make it more detailed and comprehensive. In this revision, we have included further interpretation of our findings, connected them to existing research, and discussed their implications in greater depth. We hope this makes the discussion section completer and more informative. (please go to line 249-1256, 259-263, 282, 269-272, 274-278, and 282-283)

Overall:

- The paper it is informative on knowledge of providers related to components of PAC.

Response: Thank you for your valuable feedback. we glad to hear that you found the paper informative.

- The criterion used for knowledge was very stringent (100% response as good versus all those less than 100% as poor).

Response: Thank you for your valuable comment. We chose a 100% correct response of knowledge related question as the criterion for determining knowledge because the CPAC are

essential and should not be missed. Even a single missed component can lead to significant complications and jeopardize the patient's safety. Therefore, we felt it was appropriate to define knowledgeable providers as those who correctly addressed all components of PAC, as this ensures the best care for patients. Please look at operational definition (line 160 -164).

Thank you.
